# Identification of molecularly targeted therapy-induced immunopeptidome in diffuse midline glioma (DMG)

**DOI:** 10.1016/j.neo.2026.101278

**Published:** 2026-02-04

**Authors:** Niloofar Khairkhah, Habeebah Owolabi, Ali Namvar, Mostafa M.H. Ibrahim, Seeta Nyayapathy, Richard Jones, Julie M. Rumble, Christopher E. Whitehead, Judith S. Sebolt-Leopold, Arun Everest-Dass, Stefanie Galban

**Affiliations:** a Department of Radiology, The University of Michigan Medical School, Ann Arbor, MI 48109, United States; b Institute for Biomedicine and Glycomics, Griffith University, Parklands Drive, Gold Coast, Queensland, 4222, Australia; c Immunology Services, Cayman Chemical, Ann Arbor, MI 48108, United States; d MS Bioworks, Ann Arbor, MI 48108, United States; e MEKanistic Therapeutics, Inc., Ann Arbor, MI, United States; f Department of Surgery, The University of Michigan Medical School, Ann Arbor, MI 48109, United States; g Center for Molecular Imaging, The University of Michigan Medical School, Ann Arbor, MI 48109, United States; h Rogel Cancer Center, The University of Michigan Medical School, Ann Arbor, MI 48109, United States

**Keywords:** Immunopeptidomics, Mass spectrometry, DMG, GBM, Epigenetic remodeling

## Abstract

**Introduction:**

Diffuse midline glioma (DMG) with the H3K27M mutation remains one of the most treatment-resistant pediatric brain tumors, in part due to limited antigen presentation and immune visibility. Exploring how glioma biology and therapeutic interventions influence immune recognition offers new opportunities to identify tumor-specific immune targets.

**Materials and Methods:**

We performed immunopeptidomics on human cell line derived tumor tissue for DMG and glioblastoma (GBM) and defined how MTX-241F, a selective EGFR/PI3K inhibitor, changes the tumor immunopeptidome. Immunopeptides were isolated from xenografted tumors by capturing MHC-I bound peptides followed by mass spectrometry. Comparative analyses were performed across tumor type (DMG vs. GBM) and treatment condition (vehicle vs. MTX-241F).

**Results:**

Immunopeptidomic profiling revealed tumor-specific differences in peptide repertoires between DMG and GBM. GBM tumors exhibited twice as many immunopeptides as DMG, which may be due to the distinct biology of each tumor type or may be indicative of potential HLA allotype composition. We identified highly abundant H2B1K-derived immunopeptides in DMG, suggesting that the H3K27M-driven epitranscriptome may promote turnover of other histones. MTX-241F increased the number of immunopeptides in DMG but reduced them in GBM, indicating a tumor-specific change in the immunopeptidome following EGFR/PI3K inhibition. In addition, we identified brain-enriched, HLA-A*02:01–binding and MTX-241F–exclusive immunopeptides that represent treatment-induced changes and may serve as biomarkers of therapeutic response or potential targets for CAR-T cell-based approaches.

**Discussion:**

MTX-241F changes the glioma immunopeptidome, unveiling H2B1K, brain-enriched, and treatment-induced immunopeptides as immunologically visible targets. These findings provide a rationale for integrating molecularly targeted therapy with immunotherapeutic approaches to enhance tumor recognition and treatment efficacy in DMG and GBM.

## Introduction

Diffuse midline glioma (DMG) with Lysine 27 alteration in Histone H3, previously termed diffuse intrinsic pontine glioma (DIPG), is one of the most aggressive pediatric high-grade gliomas, with overall survival rarely exceeding a year from diagnosis [1]. The recurrent H3K27M mutation profoundly alters epigenetic regulation, silences key tumor suppressor genes, and drives a highly aggressive phenotype [2]. More than 70% of DMGs harbor this mutation [3]; due to this tumor specificity and H3K27M’s role in promoting tumorigenesis [4], DMG H3K27M-altered has become the primary focus of therapeutic development. Radiotherapy remains the standard of care, as conventional chemotherapy, targeted agents, and epigenetic modulators have shown minimal clinical benefit, largely due to the tumor’s diffuse infiltrative growth and limited drug penetrance across the blood–brain barrier [5,6].

These therapeutic challenges have intensified interest in alternative approaches, such as cancer immunotherapy, which has revolutionized oncology by leveraging the immune system to detect and destroy tumor cells [7,8]. A key element of this process is the presentation of antigens by major histocompatibility complex (MHC) molecules, also known as human leukocyte antigens (HLA). These molecules on the surface of cells facilitate immune surveillance and detection. In normal cells, MHC class I molecules display peptides from intracellular proteins to cytotoxic T lymphocytes (CTLs), ensuring immune tolerance and preventing autoimmunity [9], while MHC class II molecules present extracellularly derived peptides to CD4⁺ helper T cells, coordinating adaptive immune responses [10]. In cancer cells, however, genomic instability and dysregulated pathways lead to the presentation of altered peptides on MHC-I, known as neoantigens, which serve as critical targets for immune recognition [11]. Neoantigens arise from mutations unique to cancer cells and are absent in normal tissues, providing a selective window for immune targeting with minimal risk of autoimmune toxicity [12]. However, DMGs have a low overall mutational burden compared with other types of gliomas, including adult human glioblastoma multiforme (GBM) [13], further limiting the diversity of tumor-derived neoantigens [14,15]. In DMGs, the H3K27M mutation itself generates a tumor-specific neoantigen, making it an attractive target for immune-based therapies. H3K27M-derived neoantigens have shown promise as targets for T-cell receptor (TCR)-based therapies, but the success of this approach depends on the efficient presentation of these peptides by MHC-I molecules, a process that remains poorly understood [[16], [17], [18], [19], [20]]. Recent studies have highlighted challenges in targeting H3K27M-derived immunopeptides, including inefficient epitope processing and suboptimal presentation to HLA-A*02:01 restricted CD8+ T cells [21,22]. These challenges underscore the urgent need for innovative strategies to optimize immunopeptide presentation and overcome mechanisms of tumor immune evasion.

The identification of tumor-specific neoantigens and immunopeptides has opened new avenues in immunotherapy, including the development of TCR-engineered T-cell therapies, chimeric antigen receptor (CAR) T-cell therapy and therapeutic peptide vaccines. However, despite advances in proteogenomics and mass spectrometry (MS)-based immunopeptidomics, effectively harnessing immunopeptides for therapeutic purposes remains challenging [23]. Tumor cells often develop mechanisms to evade immune detection, such as downregulating MHC expression, altering the antigen processing machinery, or presenting low-affinity peptides that fail to activate T cells [24]. The integration of MS for identifying HLA-bound peptides into experimental and computational proteogenomic approaches that enable large-scale immunopeptide identification has significantly advanced cancer immunotherapy [25]. This approach enables the precise characterization of immunopeptides produced or presented by tumors, providing critical insights into the potential vulnerabilities of cancer cells to immune-based therapies, such as CAR T-cell therapy [26,27].

Using immunopeptidomics, we characterized the MHC-I-bound peptide repertoire of H3K27M-mutant DMG in parallel with adult GBM to identify tumor-specific and treatment-induced immunopeptide changes, which may reflect the distinct biology of each tumor type but may also indicate different HLA allotype composition. HLA allotype composition determines which peptides can be presented on the cell surface and can shape both the identity and detectability of immunopeptides, thereby influencing comparative immunopeptidomic analyses across tumor types [28,29]. Our analysis allowed us to distinguish how tumor type and targeted pathway inhibition influence immunopeptide presentation. We integrated MTX-241F, a brain penetrant and selective PI3K/EGFR/DNA-PK inhibitor [30], to determine how EGFR/PI3K inhibition changes the immunopeptidome to uncover potential immunogenic peptides that could serve as targets for future immunotherapeutic strategies such as CAR T-cell therapy or therapeutic peptide vaccine strategies.

## Materials and methods

### Cell lines

H3K27M-altered pediatric diffuse midline glioma (DMG) DIPG13P* cells [31,32] and adult human glioblastoma multiforme (GBM) U87-MG cells were kindly provided by Dr. Carl Koschmann (Pediatrics, Michigan Medicine, University of Michigan) and Dr. Paul Mischel (Department of Pathology, University of California at Los Angeles, Los Angeles, CA), respectively [33]. DMG cells were cultured in working tumor stem medium (TSM) by mixing 1:1 ratio of Neurobasal-A medium (Gibco, Thermo Fisher Scientific, USA) and Dulbecco's Modified Eagle Medium F12 (D-MEM/F-12) supplemented with B27 (minus Vitamin A, Thermo Fisher Scientific, USA), Glutamax (Gibco, Thermo Fisher Scientific, USA), human epidermal growth factor (H-EGF; 20 ng/ml; Peprotech, USA), human basic fibroblast growth factor 154 aa (H-FGF-basic 154; 20 ng/ml; Peprotech), human platelet-derived growth factor (H-PDGF-AA; 10 ng/ml; Peprotech), H-PDGF-BB (10 ng/ml; Peprotech), and 0.2% Heparin solution (2 mg/mL; STEMCELL Technologies, USA) [34]. GBM cells were cultured in DMEM supplemented with 10% fetal bovine serum (FBS) and 1% penicillin-streptomycin. All cells were cultured at 37°C in a humidified incubator with 5% CO₂.

### IC₅₀ assay for MTX-241F

DIPG13P* or U87 cells were seeded at a density of 10,000 cells per well in 96-well plates and treated for 72 hours with MTX-241F, a multi-kinase inhibitor targeting PI3K, EGFR, and DNA-PK [30], or with an equimolar concentration of DMSO. Cell viability was then measured using the Cell Titer-Glo Luminescent Assay (Promega, Madison, WI) and read with an EnVision multilabel plate reader (PerkinElmer).

### Western blot

Cells were seeded 24 hours prior to treatment and incubated with MTX-241F or DMSO for 2 hours. Cells were lysed with RIPA lysis buffer (Thermo Fisher Scientific) supplemented with protease inhibitors (Complete Protease Inhibitor Cocktail, Roche) and phosphatase inhibitors (PhosSTOP, Roche). Western blotting was performed as described previously [30]. Primary antibodies against p-AKT (S473; CST #9271, RRID:AB_329825) and p-DNA-PKcs (S2056; CST #68716, RRID:AB_2939025) and HRP-conjugated secondary antibodies from Jackson ImmunoResearch were used. ECL-Plus substrate (Bio-Rad) and Bio-Rad ChemiDoc MP imager were used according to the manufacturer's recommendations.

### Xenograft models for pediatric and adult glioma

All animal protocols were approved by the University of Michigan Institutional Animal Care and Use Committee (IACUC), protocol number PRO00012162. DMG cells (DIPG13P* cells) (5 × 10^6^ in TSM base (1:1 Neurobasal-A medium and D-MEM/F-12) and a 1:1 Matrigel/cell suspension) and GBM cells (U87-MG cells) (1 × 10^6^ in 100 μl of DMEM serum-free media without Matrigel) were injected subcutaneously into the right flanks of 6–8-week-old female nude (NU/NU B/C) mice (N=8) (Charles River Breeding Labs). Tumor burden was assessed twice a week by caliper using standard tumor volume formula (Volume of tumor = d^2^ x D/2 where d is the shortest diameter and D is the longest diameter).

### In vivo treatment by MTX-241F

When flank tumors reached a volume of 100 mm^3^, mice were randomized into two experimental groups: vehicle control or MTX-241F (two per condition). The vehicle group received 10% DMSO, 2% Tween 80, 20% PEG, and 68% HPMC by oral gavage (P.O.), while the treatment group received MTX-241F at a dose of 100 mg/kg P.O. daily for 14 consecutive days. At the end of the treatment, all mice in both groups were euthanized as tumors in the vehicle group reached the study endpoint volume (∼800 mm³). Tumors were excised, snap-frozen in liquid nitrogen, and stored at -80°C. Drug toxicity was assessed by weighing mice twice a week during the 14-day treatment period.

### MHC-I expression by flow cytometry

500,000 DIPG13P* cells were fixed and permeabilized using the eBioscience Intracellular Fixation & Permeabilization Buffer Set (Thermo Fisher, Cat# 88-8824-00) with Anti-HLA Class I antibody [W6/32] (abcam, Cat# 23755), or isotype control IgG2a (CST, Cat#61656) with Goat anti-Mouse IgG (H+L) Cross-Adsorbed Secondary Antibody, Alexa Fluor™ 488 (Invitrogen, Cat#A11029) for 60 minutes each at room temperature in the dark. Samples were analyzed on ZE5#3 laser (405/488/561 nm) flow cytometer cell analyzer at the Flow Cytometry Core at the University of Michigan and analyzed using FlowJo v10.10. MHC class I expression in U87-MG cells has been extensively characterized in prior studies and was therefore not re-assessed in this study [35].

### Mouse and human MHC-I pulldowns and MHC-I ELISA

For each tumor type and treatment condition, two xenograft samples were pooled prior to lysis, lysed in Pierce IP lysis buffer at Cayman Chemical (Ann Arbor, MI, USA), and subsequently divided into mouse-depleted and non-depleted groups. For mouse-depleted samples, lysates were first incubated with M1/42 antibody–conjugated resin (cyanogen bromide-activated Sepharose [Cytiva, #17043001] conjugated with antibody at 2 mg/ml) to remove murine MHC-I molecules. The resulting supernatant was subsequently incubated overnight at 4°C with W6/32 antibody (Cayman Chemical, 20898)-conjugated resin (Protein A sepharose, Thermo, 10142) to capture human MHC-I complexes. For non-depleted samples, lysates were directly incubated with W6/32-conjugated resin overnight at 4°C to capture total MHC-I complexes. After several washes with wash buffer, bound peptides were eluted with 0.1% TFA in 0.1 M acetic acid. Eluted peptides were concentrated and desalted using solid-phase extraction (SPE) on a Waters µHLB C18 plate, lyophilized, and reconstituted in 0.1% TFA for mass spectrometry analysis. ELISA was performed pre-W6/32 and post- W6/32 immunoprecipitation to validate the successful capture of MHC class I molecules using MHC class I (human) ELISA (Cayman Chemical; #502060, Ann Arbor, MI, USA).

### Mass spectrometry

Peptides were analyzed by Liquid Chromatography–Tandem Mass Spectrometry (nano-LC/MS/MS) using a Waters NanoAcquity system coupled to a Thermo Fisher Fusion Lumos mass spectrometer by MS Bioworks (Ann Arbor, MI, USA). Peptides were loaded onto a trapping column and separated using a 75 µm analytical column packed with XSelect CSH C18 resin (Waters) at a flow rate of 350 nL/min. A 2-hour gradient was employed using mobile phases of acetonitrile and water with 0.1% formic acid. Mass spectrometry was operated in data-dependent acquisition (DDA) mode with full MS scans acquired in the Orbitrap at a resolution of 60,000 (FWHM). MS/MS scans were performed using collision-induced dissociation (CID) and electron-transfer higher-energy collision dissociation (EThcD) at a resolution of 15,000 (FWHM) across a mass range of *m/z* 300-1600. A 3-second cycle time was employed for all MS/MS scans. All raw mass spectrometry immunopeptidomics data were deposited in the PRIDE repository under accession number PXD070941.

### Peptide identification

Raw MS data files were analyzed using the immunopeptidomics workflows in FragPipe [36] and PEAKS [37] softwares. Raw spectra were searched against the Homo sapiens reference proteome (GRCh38.p14, UniProt) plus common contaminants using FragPipe v22.0 (MSFragger/Philosopher) and PEAKS DB (Bioinformatics Solutions Inc.) in a no-enzyme (nonspecific) mode appropriate for HLA-I peptides. Oxidation of methionine and deamidation of asparagine/glutamine were set as variable modifications. Precursor and fragment mass tolerances were set according to Orbitrap vendor recommendations for the Fusion Lumos acquisition. Peptide–spectrum matches (PSMs) were filtered using a target–decoy strategy to achieve a 1% false discovery rate at the PSM and peptide level and matches to decoy or common contaminant sequences were removed.

PSM-level output tables from FragPipe and PEAKS were then exported and merged. For each sample, the duplicate identifications were collapsed to generate a unified list of unique 8–11–amino acid peptides, retaining the highest-intensity identification per peptide–condition pair. All downstream analyses used this unified list of high-confidence peptides. PSM-level output tables from FragPipe and PEAKS report, for each identification, the −10lgP, precursor *m/z,* charge state, retention time, mass error, fragment-ion intensities, assigned modifications and protein accession. To annotate cross-species homologs, mouse gene names were converted to human orthologs using the HGNC Comparison of Orthology Predictions (HCOP) tool (https://www.genenames.org/tools/hcop/) [38].

### Downstream data processing and comparative analysis

We used filtered peptide lists (restricted to 8–11 amino acids) for comparative analyses across experimental conditions. Peptides with a log₂ fold change of ≥ 1 were considered differentially expressed in response to treatment.

### Cross referencing immunopeptides to FDA-approved drug targets

All identified immunopeptides were mapped to their corresponding gene symbols and cross-referenced with the Human Protein Atlas (HPA) druggable proteome dataset (https://www.proteinatlas.org/search/protein_class:FDA±approved±drug±targets) [39]. This database includes proteins classified as targets of U.S. Food and Drug Administration (FDA) approved drugs.

### Pathway analysis

Pathway analysis was performed using multiple curated databases, including Reactome (2022) [40], Elsevier Pathway Collection, MSigDB Hallmark (2020) [41], and BioCarta (2016) [42], each with its own built-in statistical model (e.g. Fisher’s exact test or binomial test) to calculate *p*-values, with *p* < 0.05 considered significant.

### Brain-enriched peptide identification

Human Protein Atlas (HPA) [39] was used to identify brain-enriched immunopeptides. The peptide list was mapped to corresponding gene symbols and cross-referenced against the HPA tissue-specific proteome and brain RNA expression datasets. Genes were classified as brain-enriched when brain mRNA expression was at least four-fold higher than in any other tissue, according to HPA’s RNA tissue specificity definitions (https://www.proteinatlas.org/humanproteome/tissue).

### Prediction of computational HLA binding

Immune Epitope Database (IEDB) (https://www.iedb.org/) [43] and MHCflurry v2.0 [44] was used to identify HLA class I binding affinity with an EL-RANK cutoff < 0.5%, corresponding to strong predicted binding affinity. A panel of 12 representative HLA Class I alleles was used to capture major A and B supertypes with global population coverage (HLA-A*01:01, A*02:01, A*03:01, A*24:02, A*26:01; HLA-B*07:02, B*08:01, B*27:05, B*39:01, B*44:02, B*58:01, B*15:01). For each tumor type (vehicle or MTX-241F), each 8–11 mer immunopeptide was assigned to the panel of 12 representative HLA Class I alleles from MHCflurry and the percentage of strong-binding immunopeptides assigned to HLA-A versus HLA-B loci was calculated. All processed datasets related to immunopeptide analyses are provided in a Master Excel file in the supplementary materials.

### Motif analysis

9-mer peptides identified from DMG or GBM tumors before and after treatment were subjected to clustering analysis using GibbsCluster 2.0 (https://services.healthtech.dtu.dk/services/GibbsCluster-2.0/), a probabilistic tool for unbiased peptide clustering. The clustering parameters were set to K = 1–3, allowing the algorithm to optimize the number of clusters by maximizing motif consistency while minimizing outliers. The sequence motifs generated from the clustering analysis were visualized using Seq2Logo 2.0 (https://services.healthtech.dtu.dk/services/Seq2Logo-2.0/), a tool for creating sequence logos.

## Results

### GBM tumors exhibit approximately twice as many MHC-I–associated immunopeptides as DMG tumors

**Fig. 1:**
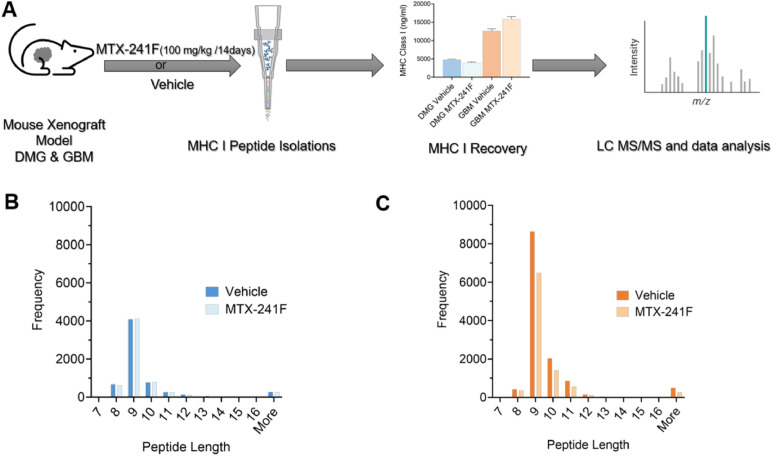
**Comparative MHC-I immunopeptidomics analysis of DMG and GBM xenografts. (A)** Schematic of the workflow. 5 × 10^6^ DIPG13P* cells or 1 × 10^6^ U87 cells were implanted into nude mice and tumor bearing mice were treated with vehicle control or MTX-241F (100 mg/kg) for 14 days by PO when tumors reached 100 mm^3^ volume. MHC-I pulldown was performed using W6/32-conjugated resin and MHC-I expression determined by ELISA prior to submitting samples for LC MS/MS analysis. **(B-C)** Profiles of peptide length distribution in treated and untreated DMG (B) and GBM (C) tumors.

To identify MHC-I immunopeptides in pediatric and adult gliomas, we collected tumors from DMG and GBM xenograft-bearing mice treated with MTX-241F (brain penetrant and selective PI3K/EGFR/DNA-PK inhibitor) [30] or vehicle, enriched MHC complexes, and analyzed them by LC–MS/MS (Fig. 1A). We identified 4458 immunopeptides in DMG and 8790 in GBM, showing that GBM displayed nearly twice as many peptides as DMG, likely due to differences in HLA allotypes, but also due possibly to tumor type–specific genetics and peptide generation. All identified peptides and peptide counts, stratified by tumor type and treatment condition, are provided in the master Excel file in the Supplementary Materials. Most identified peptides were 8–11 amino acids long (>94%) (Fig. 1B, C), consistent with canonical HLA-I ligands, and only peptides within this range were used for downstream analyses. Importantly, MTX-241F did not significantly alter peptide length distribution within either tumor type.

To assess potential host contamination, tumor lysates were subjected to mouse MHC-I depletion using the M1/42 antibody prior to W6/32 immunoprecipitation (**Fig. S1A, B**). The comparison of mouse-depleted and non-depleted samples showed that mouse peptide contamination was minimal (only 3%) (**Fig. S1A, B**). To confirm the efficiency and specificity of the MHC-I immunoprecipitation (IP), we compared the MHC-I ELISA result of pre- versus post-IP samples captured with W6/32 conjugated resins (**Fig. S1A, B**). These analyses demonstrated complete depletion of MHC-I from tumor lysates, indicating that the W6/32 resin efficiently captured MHC-I complexes. The near-complete loss of MHC class I molecules in post-IP samples confirmed the efficacy of the capture process (**Fig. S1A, B**). To precisely assess species origin, peptides were annotated based on accession numbers and categorized as human-only, mouse-only, or shared sequences identical between the human and mouse proteomes. Of the total 12,112 peptides identified, 4,564 (38%) were human-specific, 7,130 (59%) were common to both mouse and human, and 418 (3.4%) were exclusively of mouse origin, despite tumors being grown in a murine host (**Fig. S1C**). The few mouse-specific peptides likely reflect uptake of host-derived proteins by tumor cells, followed by their processing and presentation on tumor cells. After confirming species specificity, we next compared overall peptide abundance between models. As murine peptide contamination was negligible, the mouse-depleted and non-depleted datasets were merged for all subsequent analyses.

By analyzing 9-mer peptide motifs, we identified compositional differences in the immunopeptidomes of DMG and GBM. In particular, DMG samples exhibited greater motif diversity, including both classical anchor usage (P2 D/E, P9 Y/L/F) and non-canonical patterns, consistent with differences in HLA allotype composition. Importantly, MTX-241F did not induce major changes in motif composition in either DMG or GBM, only minimal changes in proportion of peptides. A third motif cluster unique to DMG contained hydrophobic and aromatic anchors not observed in GBM, indicating a distinct binding pattern potentially shaped by differences in HLA allele usage. In contrast, GBM peptides displayed more conserved motifs, dominated by classical HLA-A and HLA-B anchor residues, indicating less motif plasticity and more stable peptide-binding preferences across conditions (**Fig. S1D**).

To further verify that DMG tumor cells express MHC-I molecules on their surface, we evaluated MHC-I expression in DIPG13P* cells by intracellular flow cytometry. Nearly all DIPG13P* cells (98.5%) stained positive for HLA Class I, confirming robust MHC-I expression and supporting the validity of our immunopeptidomics approach (**Fig. S2A**). HLA Class I expression in U87-MG cells has been previously characterized [35] and was therefore not re-assessed here.

### Tumor-intrinsic pathobiology likely influences immunopeptide diversity in DMG and GBM

To ensure reliable analysis, we removed immunopeptides with missing or zero mean abundance values, yielding a high-confidence dataset representing the core MHC-I–associated repertoire. For clarity and biological interpretation, immunopeptides were represented by their corresponding source protein (see full list in master Excel file in the Supplementary Materials). We analyzed the immunopeptidomes of DMG and GBM tumors and observed differences in the number and composition of detected immunopeptides (∼6k vs. 2.3k tumor-specific immunopeptides) (**Fig. S3A**). This likely reflects differences in mutational load between DMG and GBM, but may also be due to HLA allotype composition, consistent with the higher MHC-I levels observed by ELISA in GBM tumors (**Fig. S1A-B**). A limited subset of identical peptide sequences was detected in both tumor types, that may represent putative pan-glioma immunopeptides predominantly derived from key stemness- and proliferation-associated proteins, including PTPRZ1,ID3, MCM4, VEGFA, and PRC1 (Table 1). In contrast, tumor-specific immunopeptides reflected the distinct biology of each tumor type but may also indicate different HLA allotype composition. DMG immunopeptides primarily originated from epigenetic regulators and chromatin packaging (*DNMT1, KDM1A, CHD8*), whereas GBM favored signaling and cell-cycle control (*EGFR, PDGFRB, MCM4, CDKN3*), highlighting distinct peptide landscapes between the two gliomas (Table 2). Interestingly, six peptides derived from histone H2B variants (H2B1K and H2B1A) were detected in DMG, all exhibiting exceptionally high abundance (970 × 10^7^). In contrast, H2B1K-related peptides were also identified in GBM tumors, but their abundance was markedly lower (75 × 10^7^; approximately 13-fold) than in DMG, indicating a pronounced tumor-type difference in histone-derived immunopeptides.

**Table 1:** Selected identified immunopeptides in DMG and GBM.

State	Gene	Peptides	Role	Abundance in DMG (10^7^)	Abundance in GBM (10^7^)
Stemness	**PTPRZ**	FPTEVTPHAF	OPC-like stem program marker	20.62	1
	**PHC2**	HTSAVILQL	PRC1 Subunit	2.20	3.59
	**ID3**	YILDLQVVL	Differentiation Inhibitor	2.10	172.62
Proliferation & Invasion	**MCM4**	YPQEVIPTF	DNA Replication	17.65	1.20
	VEGFA*	EVVKFMDVY	Angiogenesis	115.63	12.27
	**PRC1**	ELFEGVQKW	Epigenetic Regulation	5.48	1.22
	**CSPG4**	TMLARLASA	Cell Migration	167.19	496.33
	JUN*	MVAPAVASV	TF activator	8.53	37.18

Selected immunopeptides out of the 160 peptides detected in both DMG and GBM vehicle-treated tumor-bearing mice, grouped by biological function. Peptide abundance values are shown independently due to the distinct biology of each tumor type and potential HLA allotype composition. Selected genes were included in the table based on their biological relevance.

⁎ indicates availability of FDA-approved drug target.

**Table 2:** Comparison of selected tumor-type specific immunopeptides in DMG vehicle (out of 2363) and GBM vehicle (out of 5976).

Tumor Type	Gene	No. of Peptides	Role	Cumulative Abundance (10^7^)
**DMG**	**H2B1K**	5	Chromatin Packaging	970
	**MYCN**	5	Oncogenic TF	260
	DNMT1*	3	DNA Methylation	150
	**PGFRA**	5	Cell Signaling RTK	99
	**KDM1A**	3	Histone Demethylation	20
	HDAC5*	1	Histone Deacetylation	10
	**CHD8**	3	Chromatin Remodeler	7.8
	**H2B1A**	1	Chromatin Packaging	0.07
	**OLIG2**	1	OPC-Lineage TF	0.36
**GBM**	**CDKN3**	1	Cell Cycle	130
	**MCM4**	4	DNA-Replication Licensing	120
	**PGFRB**	2	Cell Signaling RTK	50
	EGFR*	3	PI3K-AKT Pathway Activation	4.5

Genes of a selected list of tumor-type specific immunopeptides detected in DMG (out of 2363) and GBM (out of 5976) vehicle-treated xenografts. For each gene, the number of detected immunopeptides, the cumulative immunopeptide abundance and biological function are reported. Peptide abundance values are shown independently due to the distinct biology of each tumor type and potential HLA allotype composition. Selected genes were included in the table based on their biological relevance.

⁎ indicates target availability of FDA-approved drug.

### MTX-241F changes the DMG immunopeptidome, indicative of treatment-induced effect

**Fig. 2:**
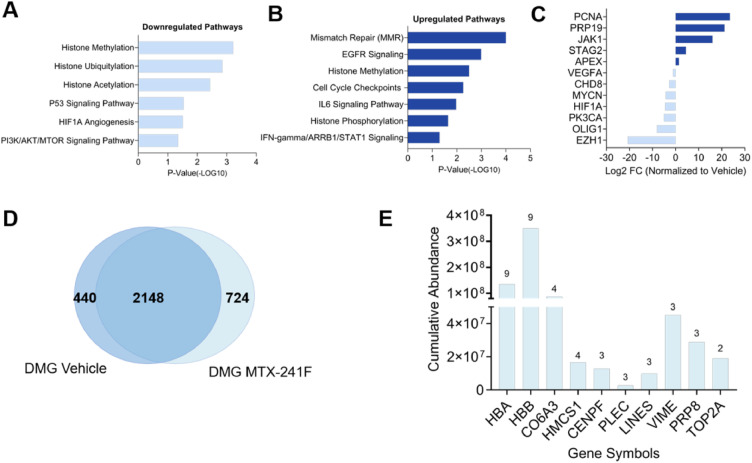
**MTX-241F induces unique immunopeptides in DMG. (A-B)** Pathway analysis of genes associated with identified immunopeptides indicating modulation by MTX-241F when compared to vehicle in DMG. **(C)** Graphical representation of downregulated and upregulated genes associated with immunopeptides identified in MTX-241F-treated DMG. **(D)** Venn diagram depicting the shared and treatment exclusive peptides in DMG treated with MTX-241F or left untreated. **(E)** Top 10 genes associated with immunopeptides identified exclusively in MTX-241F-treated DMG, ranked by cumulative abundance of MHC-I peptides.

To assess how MTX-241F alters the immunopeptidome in DMG, we compared tumors from MTX-241F–treated and vehicle-treated mice. Not surprisingly, pathway analysis revealed that MTX-241F altered multiple biological pathways, rather than having a single-pathway effect. In DMG, p53, PI3K/AKT/mTOR, histone ubiquitylation, PRC2 gene silencing and HIF signaling were downregulated, indicating effective pathway inhibition by MTX-241F (Fig. 2A). IL6 signaling was among the pathways upregulated following treatment (Fig. 2B), and the histone methylation pathway showed regulation in both directions, underscoring the complexity of chromatin reprogramming in DMG. 2,148 immunopeptides were shared between vehicle and MTX-241F in DMG, displaying mixed regulation (upregulated, downregulated, or stable) (Fig. 2C, D). The downregulation of MHC-I immunopeptides related to resistance-associated genes such as *OLIG1, EZH1, PK3CA, HIF1A*, and *MYCN* suggests that MTX-241F effectively suppresses proliferative pathways. The upregulation of immunopeptides related to checkpoint regulators like PCNA or DNA repairs (PRP19, APEX) likely indicate attempts to repair. In DMG, 724 immunopeptides were MTX-241F-exclusive, representing uniquely expressed immunopeptides that emerged in response to MTX-241F (Table 3). The number of peptides assigned to the top 10 genes was counted to determine which proteins contributed the most to the DMG MTX-241F–exclusive immunopeptidome. Several genes generated a relatively high number of peptides, whereas others contributed only a few, reflecting variability in peptide abundance (Fig. 2E).

**Table 3:** Selected DMG MTX-241F-exclusive immunopeptides out of 724.

Gene Name	Peptide Sequence	Role	Abundance (10^7^)
**PIAS1**	GKMRLTIP	SUMOlation	3.4
**ATRX**	IMDENNQWY	Chromatin Remodeler	1.41
CD276*	AQLNLIWQL	Immune Checkpoint	1.31
**LATS1**	TVMPPVAEA	Tumor Suppressor	1.2
**RAD50**	LDQELIKA	DNA Double-Strand Break Repair	0.6
**SMC1A**	NEVEIEKL	Genome Stability	0.23
**RBBP7**	SAVVEDVAW	Chromatin Assembly	0.06
**FAS**	LPKTGTVSL	Programmed Cell Death	0.036

Selected peptides out of 724 treatment (MTX-241F)-exclusive immunopeptides in DMG sorted by abundance.

⁎ indicates target availability of FDA-approved drug. Selected genes were included based on their biological relevance.

### MTX-241F changes the GBM immunopeptidome, indicative of treatment-induced effect

**Fig. 3:**
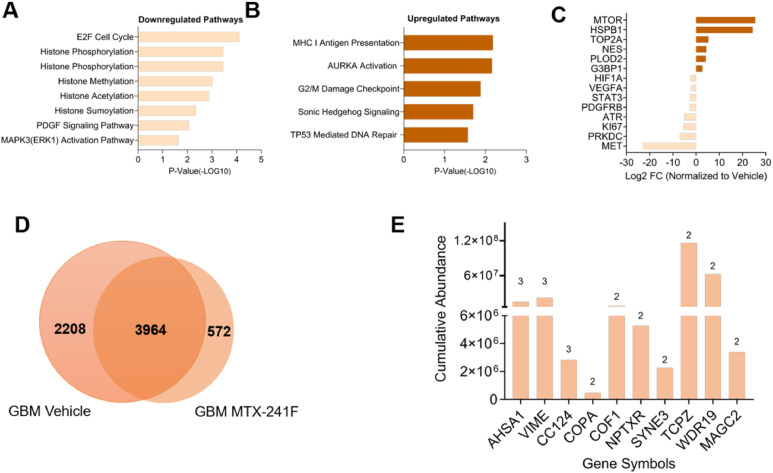
**MTX-241F induces unique immunopeptides in GBM. (A-B)** Pathway analysis of genes associated with identified immunopeptides indicating modulation by MTX-241F when compared to vehicle in GBM. **(C)** Graphical representation of downregulated and upregulated genes associated with immunopeptides identified in MTX-241F-treated GBM. **(D)** Venn diagram depicting the shared and treatment exclusive peptides in GBM treated with MTX-241F or left untreated. **(E)** Top 10 genes associated with immunopeptides identified exclusively in MTX-241F-treated GBM, ranked by cumulative abundance of MHC-I peptides.

To assess how MTX-241F alters the immunopeptidome in GBM, we compared tumors from MTX-241F–treated and vehicle-treated mice. In GBM, MTX-241F modulated a distinct set of pathways in comparison with DMG, with decreased representation of chromatin regulation and histone modification (Fig. 3A) and increased representation of cell cycle and check point regulation peptides (Fig. 3B). In GBM, MTX-241F was associated with decreased PDGF and reduced MAPK3/ERK1 activation, consistent with upstream EGFR/PI3K pathway inhibition. Conversely, the treatment enhanced antigen presentation and DNA-repair pathways and elevated AURKA/mitotic and Sonic Hedgehog (SHH) signaling (Fig. 3B). These changes reflect both therapeutic activity (improved immunopeptide presentation) and adaptive stress responses (AURKA, SHH). In GBM, 3964 peptides were shared between vehicle and MTX-241F groups, displaying mixed regulation like what we observed in DMG (Fig. 3C, D) and 572 peptides were MTX-241F-exclusive (Fig. 3E and Table 4).

**Table 4:** Selected GBM MTX-241F-exclusive immunopeptides out of 572.

Gene Name	Peptide Sequence	Role	Abundance (10^7^)
**PRKDC**	TVLEKVYEL	DSB Repair	2.19
**HSP7C**	LLDVTPLSL	Chaperone	1.33
**PSMD5**	SQDPTMIGV	proteasome regulator	1.07
**PRKRA**	SEIAKEQGF	IFN Signaling	0.7
**WEE1**	VLGQHSHVV	G2/M Checkpoint	0.15
**IFIT2**	RLSDVQIYV	Type I/II IFN Response Marker	0.06
**PSMD7**	QEFVKAFYL	Antigen Processing	0.02
**PSMD1**	SVIQNLRTV	Generation for MHC-I	0.02

Selected peptides of the 572 treatment exclusive peptides, which were sorted by peptide abundance and indication of biological function. Selected genes were included based on their biological relevance.

### Identification of brain-enriched, HLA-A*02:01–bound immunopeptides in MTX-241F–treated DMG and GBM tumors

To prioritize physiologically relevant candidates from the MTX-241F–treated immunopeptide pool in both tumor types, we focused on immunopeptides that are brain-enriched and predicted to bind HLA-A*02:01, a widely targeted HLA molecule in immunotherapy (Table 5). Of the 2872 immunopeptides identified in treated DMG, 77 were brain-enriched. Similarly, of the 4536 immunopeptides identified in treated GBM, 80 were brain-enriched. We observed only minor percentage changes (≤3%) in HLA-A– and HLA-B–restricted immunopeptides between vehicle and MTX-241F in DMG and GBM tumors (Fig. S5A–B). These results indicate that MTX-241F may change peptide presentation without altering the predicted HLA composition.

**Table 5:** Selected brain-enriched peptides identified in DMG (77 out of 2872) and GBM (80 out of 4536 total), predicted to bind HLA-A*02:01.

**DMG**				**GBM**			
Gene	Peptide	Role	Strong Binders HLAs	Gene	Peptide	Role	Strong Binders HLAs
**SMG1**	FTADFVRQL	DNA Damage Response	HLA-A*02:01,HLA-B*27:05,HLA-B*58:01	**PREX1**	SLAEVAGLQV	RTK Trafficking	HLA-A*02:01
**PI4KA**	EAANFIMKV	RTK Trafficking	HLA-A*02:01	**DDX1**	ILAPTVQEL	Supports Replication Stability	HLA-A*02:01,HLA-A*24:02,HLA-B*08:01,HLA-B*27:05
**NOVA1**	FIAEKIREM	Neuron-Specific Splicing Factor	HLA-A*02:01,HLA-B*07:02,HLA-B*08:01	AHR*	QQDPQQYNV	Immunoregulatory TF	HLA-A*02:01
**LPP**	TAMDQVFHV	Invasion/Migration	HLA-A*02:01	TOP2A*	KLDETGNSL	Replication	HLA-A*02:01,HLA-B*07:02,HLA-B*08:01,HLA-B*27:05
**FGD1**	FPADVVHGI	Cytoskeletal Remodeling	HLA-A*02:01,HLA-B*07:02,HLA-B*08:01,HLA-B*35:01	**NSD3**	AQWDIGIAHA	NOTCH Signaling Pathway	HLA-A*02:01
**RPTOR**	SAHEKLYSL	mTOR Axis	HLA-A*02:01,HLA-B*07:02,HLA-B*08:01	**CAV1**	AVGKIFSNV	Cytoskeletal Remodeling	HLA-A*02:01
**DDX47**	EVMMLTERV	Ribosome Biogenesis	HLA-A*02:01	**MGLL**	KIYEGAYHV	Reprograms Lipid Metabolism	HLA-A*02:01,HLA-A*03:01
**APC**	TASKLPPPV	WNT signaling	HLA-A*02:01	**CIC**	ALQELTQMV	Tumor Suppressor	HLA-A*02:01
**NCOR2**	EVSEIIDGL	HDAC3 recruiter	HLA-A*02:01	**BST2**	LLQQELTEA	Type-I IFN/Stress Signaling	HLA-A*02:01
**ARSK**	ETFKNEHKV	Lysosomal Arylsulfatase	HLA-A*02:01	**DDR1**	YLQVDLQRL	ECM-Coupled Survival	HLA-A*02:01

Seventy-seven immunopeptides in treated DMG and 80 in treated GBM were found to be brain-enriched using the Human Protein Atlas database. Selected genes were included based on their biological relevance. * indicates availability of FDA-approved drug target.

### MTX-241F differentially regulates immunopeptides detected in both DMG and GBM

**Fig. 4:**
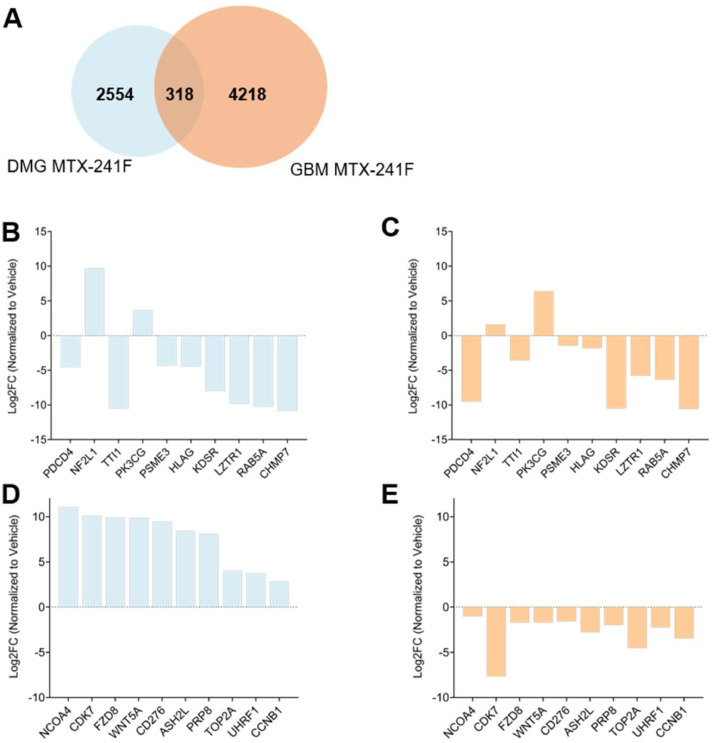
**Identification of peptides induced by MTX-241F in DMG and GBM. (A)** Venn diagram depicting immunopeptides detected following MTX-241F in DMG and GBM, including peptides detected in both datasets and those detected in only one tumor type. **(B-C)** Log2 fold change (normalized to vehicle) of selected immunopeptides detected in both tumor types despite differences in tumor biology and HLA allotype composition, shown for DMG (B) and GBM (C), demonstrating similar regulation patterns following MTX-241F. **(D-E)** Log2 fold change (normalized to vehicle) of selected immunopeptides detected in both tumor types despite differences in tumor biology and HLA allotype composition, shown for DMG (D) and GBM (E), demonstrating different regulation patterns following MTX-241F.

To examine how MTX-241F regulates the immunopeptidome across tumor types, we first identified the immunopeptides that were detected in both DMG and GBM datasets (Fig. 4A). A small number of peptide sequences (318 peptides) were detected in both tumor types, whereas the majority were detected in either DMG (2,554 peptides) or GBM (4,218 peptides), indicating tumor-specific immunopeptidome modulation upon treatment exposure. To assess whether peptides detected in both tumor types exhibited similar or divergent treatment-associated behavior, we classified them into three response categories: concordant (regulated in the same direction), discordant (opposite regulation), and non-responsive to MTX-241F. Most immunopeptides showed discordant behavior between DMG and GBM (80.5%), with only a small fraction being concordant (16.98%) or non-responsive (2.52%) (**Fig. S6A**). While some peptides, such as PDCD4 and PSME3, displayed concordant regulations between DMG and GBM, others including CD276 displayed opposite regulation (Fig. 4B-E). Collectively, these findings demonstrate that MTX-241F elicits highly tumor-specific alterations in immunopeptidomes, reflecting differential adaptive responses in DMG and GBM to EGFR/PI3K inhibition, shaped by the distinct biology of each tumor type and potentially, HLA allotype composition. This is supported by our *in vitro* IC50 assays using DIPG13P* and U87 cell lines, which showed higher sensitivity of DIPG13P* cells (IC₅₀ = 9.97 µM) compared to U87 cells (IC₅₀ = 14.44 µM) (**Fig. S4A**). Western blot analysis further confirmed inhibition of the DNA-PK and AKT signaling axis, as indicated by reduced phosphorylation of DNA-PK (Ser2056) and AKT (Ser473) following treatment (**Fig. S4B**).

### MTX-241F reshapes immunopeptide repertoires in a tumor-specific manner

**Fig. 5:**
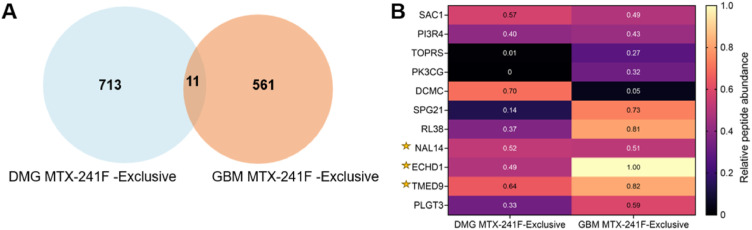
**Identification of MTX-241F–exclusive immunopeptides detected in both pediatric and adult gliomas. (A)** Venn diagram of immunopeptides between DMG and GBM, which are exclusive to MTX-241F in either tumor. **(B)** Heatmap of immunopeptide abundance of the 11 shared peptides (log₁₀-transformed, min–max scaled to the [0,1] range). Star indicates potential to bind HLA-A02:01.

To identify immunopeptides exclusively detected in response to MTX-241F, we analyzed the MTX-241F–exclusive immunopeptidomes in DMG and GBM (Fig. 5A). Only a small number of peptide sequences (11 immunopeptides) were detected in both tumors (Fig. 5B), indicating that MTX-241F alters immunopeptidomes differently. Among these immunopeptides, ECHD1, TMED9 and NAL14 are predicted to bind HLA-A*02:01 (**Table S1**). Overall, these findings suggest that MTX-241F changes the repertoire of presented immunopeptides in a tumor-specific manner.

## Discussion

Immunotherapy has revolutionized oncology by utilizing the immune system to target and eliminate tumor cells [45]. A fundamental component of this immune response is antigen presentation by MHC class I molecules, which enables cytotoxic T cells to detect and target malignant cells. The identification and characterization of tumor-specific neoantigens provide opportunities to develop precision immunotherapies tailored to each tumor’s molecular profile [11]. However, challenges such as immune evasion and inefficient antigen presentation remain barriers to effective treatment [46].

DMG, especially with the H3K27M mutation, exemplifies these challenges, as it is considered an immune cold tumor [47]. While the H3K27M mutation drives aggressive tumor phenotypes, it also presents opportunities for targeted therapies [4]. There are currently 89 clinical trials testing therapies for DMG, with an increasing number evaluating immunotherapy strategies including vaccine-based and CAR T-cell therapies (clinicaltrials.gov). CAR T-cell therapy has shown great promise in targeting tumor-specific antigens, though its success depends heavily on identifying robust and tumor-specific neoantigens [48]. One significant challenge in targeting the H3K27M mutation is its insufficient presentation by MHC class I molecules. Wang et al. [21] investigated the feasibility of using CAR T-cells to target an HLA-A*02:01-restricted H3K27M epitope in DMG. While CAR T-cells showed specific binding to cells presenting the H3K27M peptide, they were unable to identify and eliminate H3K27M-positive tumor cells derived from patients. This lack of efficacy was attributed to the absence of detectable levels of H3K27M-derived peptides, specifically the RMSAPSTGGV peptide (H3K27M^26–35^), on the surface of these cells, suggesting that the low abundance of this neoantigen limits its utility as an effective immunotherapy target in DMG. Conversely, strategies to enhance antigen presentation through MHC class I molecules have shown promise, as demonstrated by Tailor et al. [49], that ionizing radiation upregulates key regulators of antigen processing and presentation, leading to a broader repertoire of peptides displayed by MHC class I molecules. This suggests that combining radiotherapy with immunotherapeutic approaches could improve the visibility of tumor antigens to the immune system, potentially overcoming some mechanisms of immune evasion.

Here, we focus on uncovering the immunopeptidome in DMG tumors by leveraging advanced immunopeptidomics to characterize MHC-I repertoire and identify potential targets for precision immunotherapy. We extended our analysis to GBM tumors to characterize MHC-I–bound immunopeptides and identify GBM-specific candidates. The addition of GBM allowed us to better understand differences in immunopeptide presentation between pediatric and adult gliomas. Since DMG is rare [50], identifying immunopeptides that appear in both pediatric and adult gliomas may help prioritize candidates with broader therapeutic relevance, even though known differences in tumor-type or HLA allotype composition exist. Similar approaches have been used in prior studies to identify immunopeptides across different tumor types [51,52]. Using GBM allowed us to assess whether the changes observed in DMG were unique to the H3K27M-mutant context or indicative of general response to MTX-241F. This approach highlights how tumor origin and genetic background influence treatment response and immune visibility across glioma.

Regardless of treatment status, GBM exhibited a higher number of immunopeptides than DMG, which likely reflects the distinct biology of each tumor type, such as mutational load or tumor mutational burden (TMB) [53], but may also indicate potential differences in antigen processing and presentation capacity due to HLA allotype composition. GBM cells are characterized by high metabolic and proliferative activity [54], a state often associated with increased protein turnover and enhanced peptide presentation that may support a broader MHC-I repertoire. This may be influenced by the higher number of somatic mutations typically observed in GBM [55]. In contrast, the lower number of immunopeptides in DMG likely reflects its less proliferative nature and may also be influenced by its lower somatic mutation burden [13]. The presence of histone-derived immunopeptides, including multiple fragments with high abundance from H2B1K, suggests that chromatin remodeling and nucleosomal instability may influence immunopeptide turnover in DMG. These findings highlight that each tumor type exhibits a distinct immunopeptidome defined by its pathobiology and genetic makeup but may also indicate HLA allotype differences, with GBM primarily influenced by growth and signaling pathways and DMG by epigenetic dysregulation.

We detected H2B1K-related immunopeptides ((UniProtKB/Swiss-Prot: O60814) *HIST1H2BK*) with high abundance in DMG. Notably, elevated *HIST1H2BK* expression has been associated with poor prognosis in low-grade glioma and other cancers [[56], [57], [58]]. The specific presence of highly abundant H2B1K in DMG may suggest a potential link between the H3K27M mutation and histone modification crosstalk. Several studies have noted crosstalk between H2B and H3 modifications, suggesting potential interplay between these histones in chromatin remodeling [[59], [60], [61], [62]]. While our data did not explicitly confirm direct interactions between H2B1K and H3, the presence of multiple H2B1K-derived immunopeptides in DMG supports the possibility of indirect regulatory relationships between these histones. Histone modifications often interact through crosstalk, where one modification influences the establishment or recognition of another. For instance, histone H2B ubiquitination can promote di- and tri-methylation of histone H3 at Lys4 and Lys79, demonstrating how crosstalk between different marks contributes to gene regulation [59,61,63,64]. H2B1K-related peptides were also detected in GBM, but their abundance was roughly 13-fold lower, reflecting the distinct biology of each tumor type and potential HLA allotype composition, and indicating that chromatin-derived immunopeptides are far less pronounced in GBM than DMG. This preferential enrichment in DMG suggests that H2B1K immunopeptides (including ESYSVYVYK, FVNDIFERI, FVNDIFERIA, FVNDIFERIAG, LLLPGELAK) could serve as biomarkers of chromatin remodeling and potential immunotherapeutic targets. Although we observed total loss of ESYSVYVYK and LLLPGELAK related to H2B1K in DMG following MTX-241F treatment, the low turnover rate of histones ensures that sufficient levels of other immunopeptides, such as FVNDIFERI, FVNDIFERIA, and FVNDIFERIAG, remain stable on tumor cells. Interestingly, this could serve as a strategic advantage in the development of molecularly targeted therapy in combination with CAR-T cells recognizing treatment-induced immunopeptides. The stability of histones ensures that histone-derived immunopeptides remain present on tumor cells, even following molecularly targeted therapies. This prolonged presence of the peptide could make it an ideal target for immunotherapy approaches. In this context, immunotherapy could be paired with MTX-241F to enhance overall therapeutic response. While MTX-241F disrupts tumor growth, H2B1K immunopeptides would remain accessible for immune targeting, due to their low turnover.

Immunopeptidomics profiling following molecularly targeted therapy provides a valuable approach to investigating changes in immunopeptide presentation across different tumors. We have demonstrated that MTX-241F markedly reduced tumor growth in DMG [30], but showed only modest effects in GBM. This difference likely reflects the greater intrinsic dependence of H3K27M-mutant cells on EGFR/PI3K pathway signaling, making it more vulnerable to MTX-241F inhibition. Interestingly, this molecular selectivity was also reflected in the immunopeptidome profiles. MTX-241F increased the number of peptides identified in DMG but reduced it in GBM, underscoring tumor–specific change of the immunopeptidome. The increase in the DMG immunopeptidome likely reflects enhanced protein turnover and peptide processing following EGFR/PI3K inhibition. In contrast, lower GBM immunopeptidome likely indicates that MTX-241F induced lower proteome change in this tumor type. MTX-241F profoundly changes the immunopeptidome in both DMG and GBM, revealing treatment-induced remodeling.

The distinct immunopeptidome responses to MTX-241F in DMG and GBM underscore that EGFR/PI3K inhibition carries different biological consequences across these gliomas. We identified several immunopeptides in both DMG and GBM following MTX-241F treatment, despite differences in tumor type and potential HLA allotype composition. This finding suggests that MTX-241F may affect common EGFR/PI3K-associated signaling pathways in both tumor types. Among these identified peptides, some exhibited similar regulations, whereas others showed divergent regulations between tumor types. The discordant regulation implies that each tumor adapts uniquely to treatment, reshaping its immunopeptidome through distinct compensatory pathways. The small set of MTX-241F–exclusive peptides found in both DMG and GBM, notably those with binding affinity to HLA-A*02:01, suggests that EGFR/PI3K inhibition triggers conserved pathways that can be exploited therapeutically. We hypothesize that targeting these treatment-induced immunopeptides could potentially synergize with immunotherapies by enhancing peptide presentation that emerges following EGFR/PI3K inhibition.

In conclusion, this study demonstrates that MTX-241F modulates the DMG and GBM immunopeptidome, revealing both tumor-intrinsic and treatment-induced immunopeptides with potential application in future immunotherapeutic approaches. H2B1K-derived immunopeptides are compelling candidates, as their stability and high abundance in DMG suggest that histone turnover sustains peptide availability within H3K27M-mutant tumors. In parallel, brain-enriched, HLA-A*02:01–binding immunopeptides could be exploited for immunotherapeutic approaches such as CAR-T cell therapy. Moreover, MTX-241F–exclusive immunopeptides highlight the emergence of treatment-induced, immunologically visible targets that may function as biomarkers of MTX-241F response. Our findings suggest the potential for integrating molecularly targeted therapies with CAR-T cells or peptide-based vaccines directed against such immunopeptides in DMG and GBM. Despite these insights, this study has several limitations. From an experimental perspective, immunopeptidomic analyses were performed on a limited number of samples. To address heterogeneity in flank xenograft models, two tumors within each treatment condition were pooled prior to MHC-I pull down and subsequently divided into mouse-depleted and non-depleted groups for mass spectrometry analysis. In addition, biological and antigen-presentation heterogeneity may influence the results, as differences in HLA allotype composition between tumor types could affect immunopeptide detection and comparison across groups. U87 cells are a homozygous HLA-A, -B, and -C cell line expressing HLA-A*02:01, HLA-B*44:02, and HLA-C*05:01 [65]*,* whereas comprehensive HLA typing information is not available for DIPG13P* cells; however, motif analysis suggests the presence of multiple HLA allotypes distinct from U87, which may contribute to differences in peptide presentation and detection. Therefore, we acknowledge that some observed differences might reflect mass spectrometry detectability biases associated with different HLA allotype composition. In future work, we will validate these findings in larger cohorts and apply enhanced detection and analytical approaches to further characterize these peptides and assess their immunotherapeutic potential. Collectively, these results establish a framework in which molecularly targeted therapies not only suppress oncogenic signaling but also modulate the immunopeptidome, revealing treatment-associated changes. This supports the use of immunopeptidomics to guide combination strategies with FDA-approved targeted agents currently under investigation for recurrent DMG.

## Authors’ disclosures

C.E.W. has ownership interest in and is an employee of MEKanistic Therapeutics, Inc. and J.S.L. has ownership interest in and is a consultant for MEKanistic Therapeutics, Inc

## Funding

This work was supported by the National Institute of Neurological Disorders and Stroke (NIH) Grant 1R01NS13151501 (SG) and the ChadTough Defeat DIPG Foundation (SG). MTX-241F was generously provided by MEKanistic Therapeutics, Inc. A.E.D gratefully acknowledges support from Advance Queensland Industry Fellowship.

## Data availability

The mass spectrometry immunopeptidomics raw data generated in this study have been deposited in the PRIDE repository (http://www.ebi.ac.uk/pride) under the accession number **PXD070941.**

All processed datasets corresponding to Fig. 1, Fig. 2, Fig. 3, Fig. 4, Fig. 5 are provided in the Master Excel file in the supplementary materials.

## CRediT authorship contribution statement

**Niloofar Khairkhah:** Writing – review & editing, Writing – original draft, Methodology, Investigation, Formal analysis, Data curation. **Habeebah Owolabi:** Investigation, Formal analysis, Data curation. **Ali Namvar:** Methodology, Formal analysis, Data curation. **Mostafa M.H. Ibrahim:** Methodology, Data curation. **Seeta Nyayapathy:** Methodology, Data curation. **Richard Jones:** Methodology, Data curation. **Julie M. Rumble:** Methodology, Data curation. **Christopher E. Whitehead:** Methodology, Data curation. **Judith S. Sebolt-Leopold:** Methodology, Data curation. **Arun Everest-Dass:** Writing – review & editing, Methodology, Funding acquisition, Data curation. **Stefanie Galban:** Writing – review & editing, Writing – original draft, Validation, Supervision, Methodology, Formal analysis, Conceptualization.

## Declaration of competing interest

The authors declare the following financial interests/personal relationships which may be considered as potential competing interests:

C.E.W. has ownership interest in and is an employee of MEKanistic Therapeutics, Inc. J.S.L. has ownership interest in and is a consultant for MEKanistic Therapeutics, Inc.

## Appendix Supplementary materials

Image, application 1

Image, application 2

Image, application 3
