# Supplementary material for: Identification of molecularly targeted therapy-induced immunopeptidome in diffuse midline glioma (DMG)
Source: Neoplasia. 2026 Feb 4;73:101278. doi: 10.1016/j.neo.2026.101278 (PMC12918168; doi:10.1016/j.neo.2026.101278)
Supplement: Supplementary file 2 [file mmc2.docx]

*Supplemental Table 1: Common immunopeptides identified in DMG and GBM MTX-241F-exclusive (out of 11) predicted to bind HLA-A*02:01*

| **Gene** | **Peptide** | **Role** | **Strong Binders HLAs** |
| --- | --- | --- | --- |
| **NAL14** | LGDGGVKL | Regulatory role in innate immune system | HLA-B51:01, HLA-B07:02, HLA-A02:01, HLA-B08:01, HLA-A01:01, HLA-A24:02, HLA-B58:01, HLA-B44:02, HLA-B15:01, HLA-A03:01, HLA-A26:01 |
| **TMED9** | ILQTLILV | Protein Trafficking | HLA-A02:01, HLA-B51:01, HLA-B08:01, HLA-B15:01, HLA-A24:02, HLA-A03:01, HLA-A01:01, HLA-A26:01, HLA-B44:02 |
| **NAL14** | VLGDGGVK | Regulatory role in innate immune system | HLA-A03:01, HLA-A02:01, HLA-B15:01, HLA-A01:01, HLA-B07:02, HLA-B44:02, HLA-A24:02, HLA-A26:01, HLA-B08:01, HLA-B58:01 |
| **TMED9** | SILQTLIL | Protein Trafficking | HLA-B08:01, HLA-A02:01, HLA-B07:02, HLA-B15:01, HLA-B58:01, HLA-A03:01, HLA-A24:02, HLA-A01:01, HLA-A26:01, HLA-B44:02 |
| **ECHD1** | QLLEKVIE | Fatty acid Biosynthesis | HLA-A02:01, HLA-B08:01, HLA-A03:01, HLA-B15:01, HLA-B44:02, HLA-A24:02, HLA-B07:02, HLA-A01:01, HLA-A26:01, HLA-B58:01 |
