# Supplementary material for: Identification of molecularly targeted therapy-induced immunopeptidome in diffuse midline glioma (DMG)
Source: Neoplasia. 2026 Feb 4;73:101278. doi: 10.1016/j.neo.2026.101278 (PMC12918168; doi:10.1016/j.neo.2026.101278)
Supplement: Supplementary file 3 [file mmc3.pdf]

Supplemental Figures

Khairkhah et al.
Identification of molecularly targeted therapy-induced immunopeptidome in diffuse midline glioma (DMG)

Supplemental Figure 1

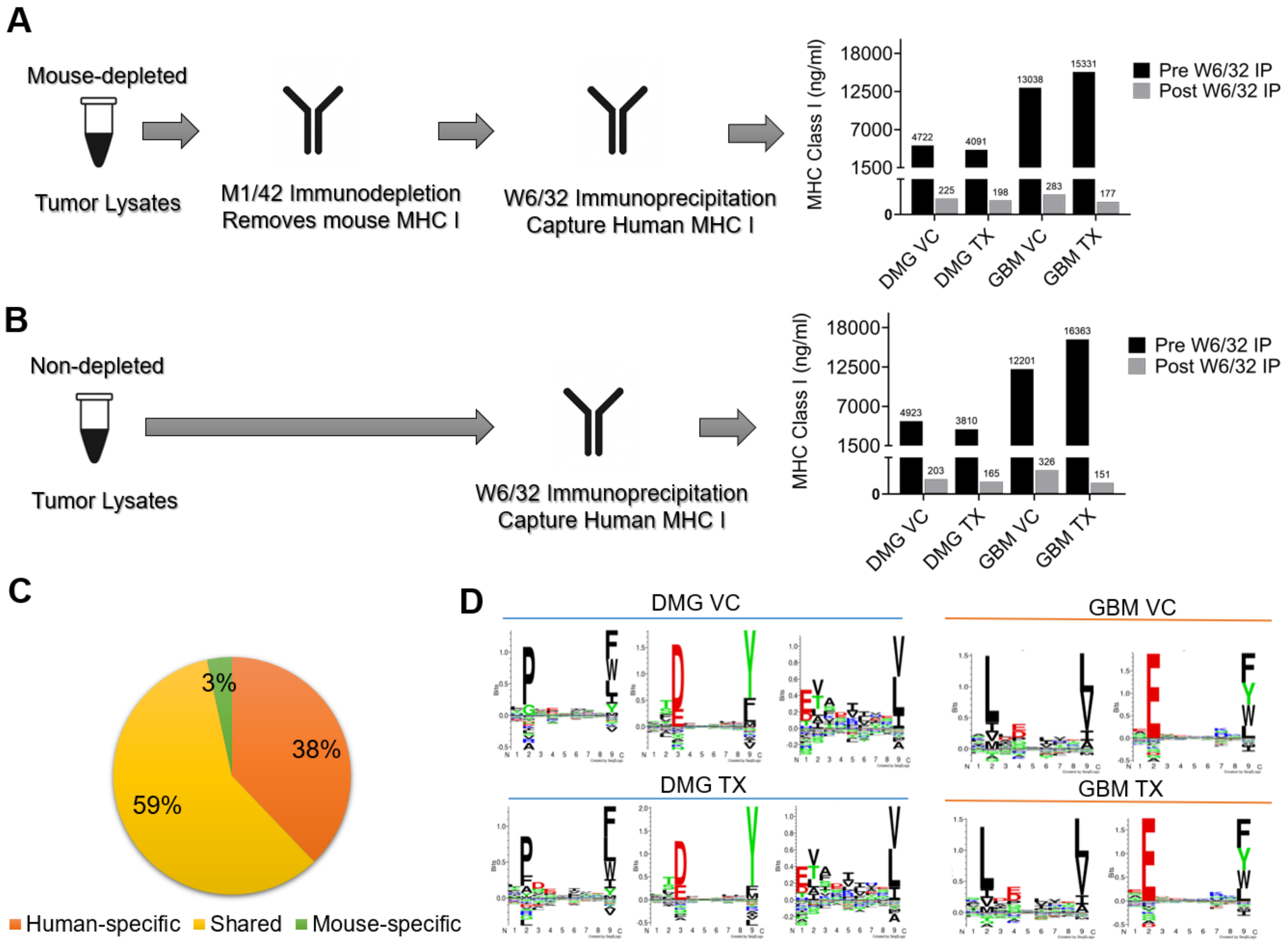

Supplemental Figure 1. Efficient MHC-I molecule capture and characterization of peptide origin in DMG and GBM xenografts. (A) Schematic of the immunopeptidomics workflow showing murine MHC-I removal by M1/42-conjugated resin prior to W6/32-mediated capture of human MHC-I associated peptides. **(B)** MHC-I capture from tumor lysates using W6/32-conjugated resin, followed by ELISA of MHC-I before and after W6/32 capture. **(C)** Distribution of identified peptides based on species specificity. Only 3% of peptides were mouse-derived. **(D)** Motif analysis of MHC-I–bound immunopeptides from DMG and GBM before and after treatment with MTX-241F. These logos highlight the most conserved amino acid residues at specific positions across the peptides in each cluster.

Supplemental Figure 2

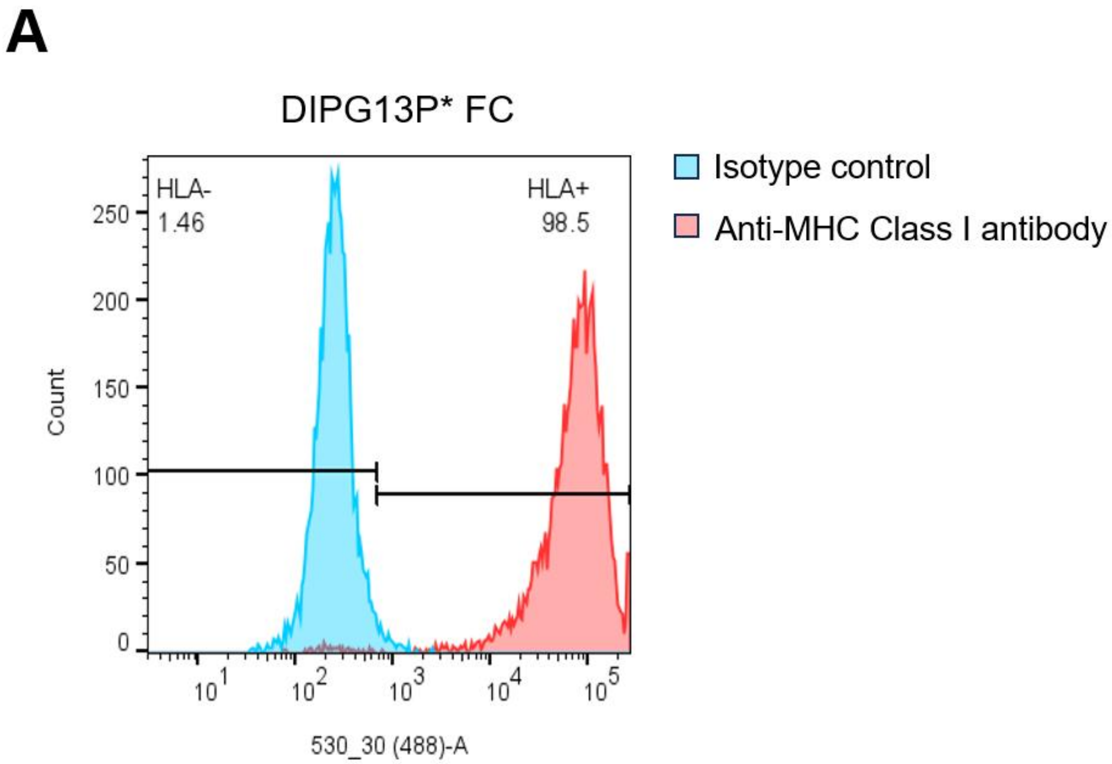

Supplemental Figure 2. Flow cytometry analysis of MHC-I expressed on DIPG13P*. (A) Representative histogram showing MHC-I-negative (IgG isotype control) and MCH I-positive (MHC-I antibody) cell populations. 500,000 DIPG13P* were fixed, permeabilized and stained. More than 98% of cells expressed MHC-I on their surfaces. MHC class I expression in U87-MG cells has been extensively characterized in prior studies and was therefore not re-assessed in this study (35).

Supplemental Figure 3

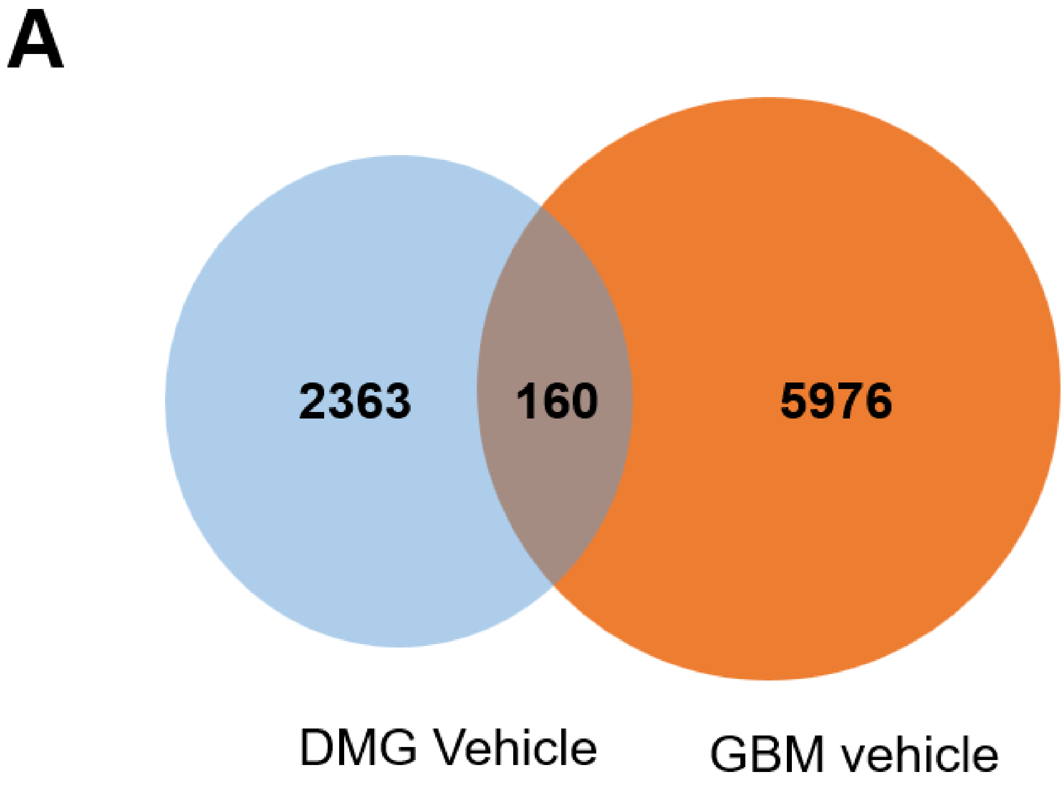

Supplemental Figure 3. 8-11 mer MHC-1 immunopeptides in pediatric (DMG) and adult glioma (GBM). **(A)** Venn diagram illustrating the distribution of shared and unique 8–11 mer peptides in vehicle treated DMG and GBM tumor xenografts.

Supplemental Figure 4

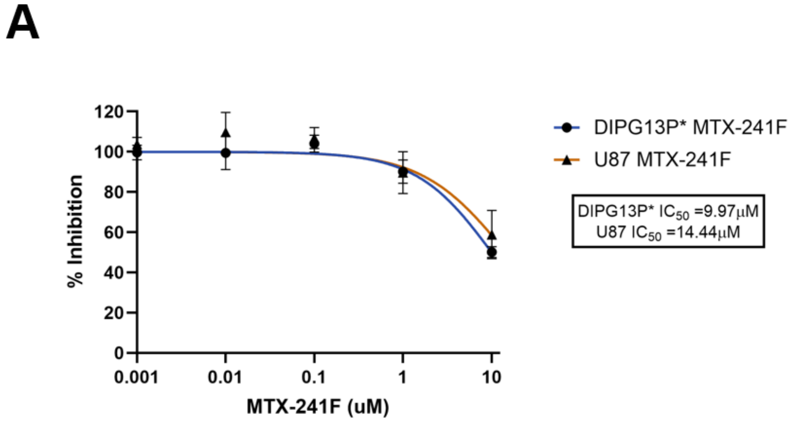

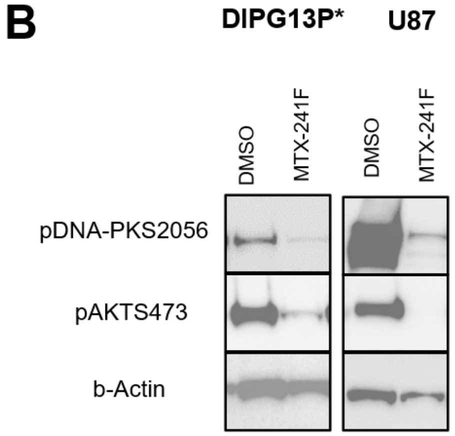

Supplemental Figure 4. MTX-241F activity in DMG and GBM cell lines. (A) In vitro proliferation assay (CellTiter-Glo) of DIPG13P* and U87 cells treated with increasing concentrations of MTX-241F (0.001–10 µM) for 72 hours. Data represent mean ± SEM from four independent experiments performed in triplicate. **(B)** Western blot analysis of phospho-DNA-PK (Ser2056) and phospho-AKT (Ser473) in DIPG13P* and U87 cells treated with MTX-241F (1 µM, 2 h) or DMSO. β-Actin served as a loading control.

Supplemental Figure 5

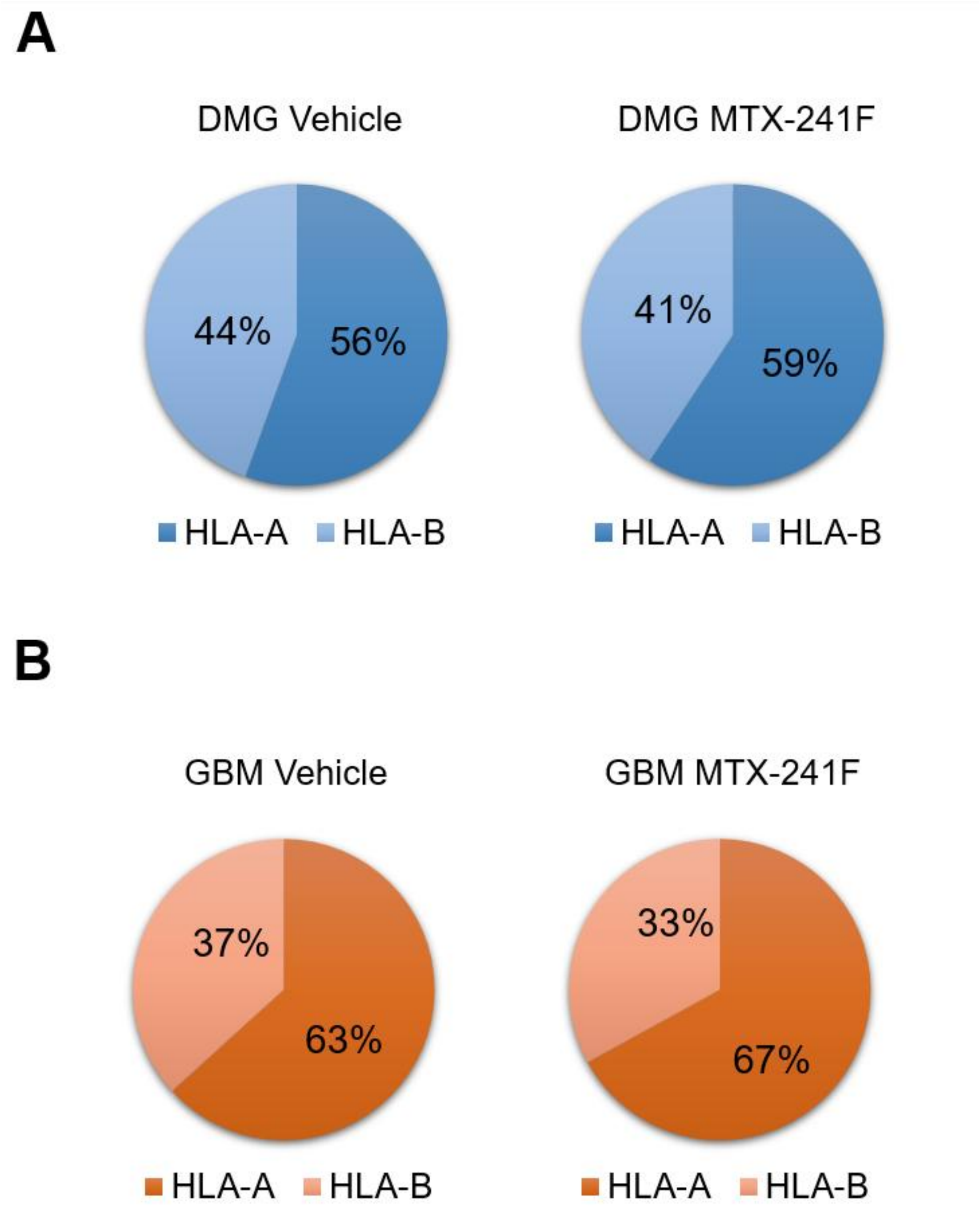

Supplemental Figure 5. Predicted HLA class I composition in DMG and GBM following treatment with MTX-241F. (A-B) Pie charts showing the percentage of immunopeptides predicted to bind HLA-A versus HLA-B alleles in DMG **(A)** and GBM **(B)** tumors under vehicle and MTX-241F conditions.

Supplemental Figure 6

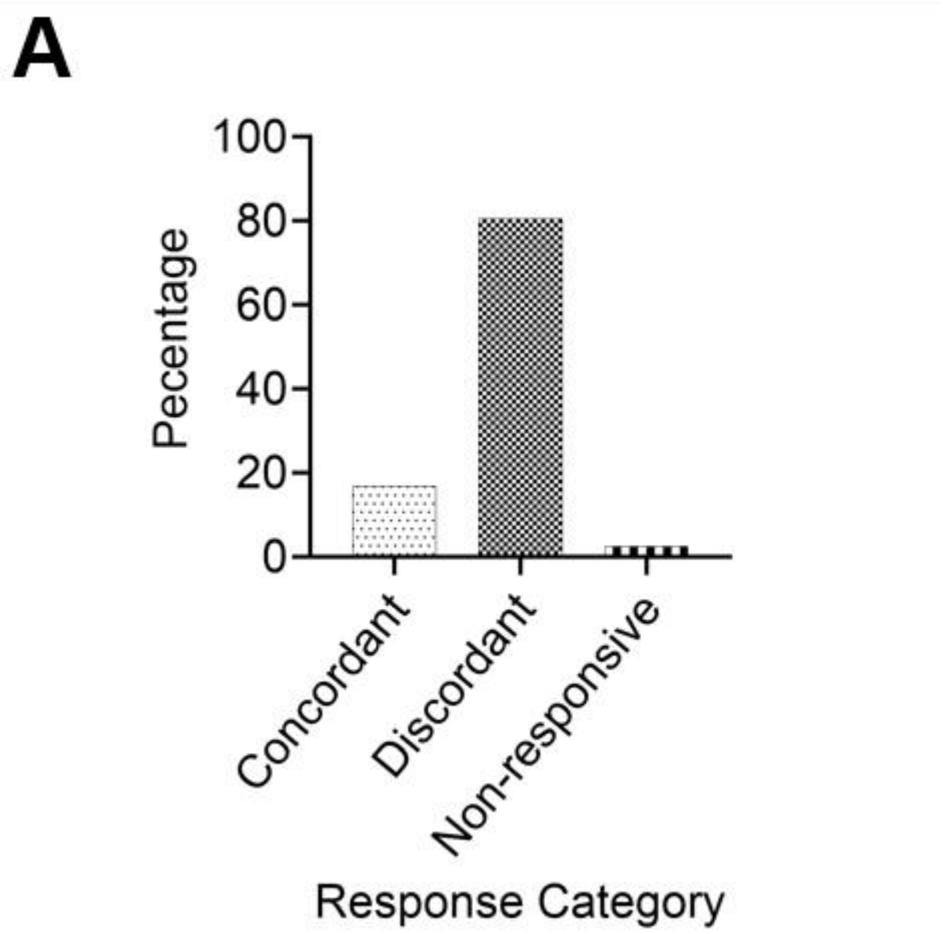

Supplemental Figure 6. Distribution of immunopeptide response following MTX-241F treatment. (A) Bar graph depicting percentage of concordant or discordant immunopeptides of the 318 similar peptides identified in both DMG and GBM.
